# Divergent drivers of the spatial variation in greenhouse gas concentrations and fluxes along the Rhine River and the Mittelland Canal in Germany

**DOI:** 10.1007/s11356-024-33394-8

**Published:** 2024-04-22

**Authors:** Ricky Mwangada Mwanake, Hannes Klaus Imhof, Ralf Kiese

**Affiliations:** Karlsruhe Institute of Technology, Institute for Meteorology and Climate Research, Atmospheric Environmental Research (IMK-IFU), Kreuzeckbahnstrasse 19, 82467 Garmisch-Partenkirchen, Germany

**Keywords:** N_2_ fluxes, Harbors, External inputs, Metabolism rates, N fixation, Denitrification

## Abstract

Lotic ecosystems are sources of greenhouse gases (GHGs) to the atmosphere, but their emissions are uncertain due to longitudinal GHG heterogeneities associated with point source pollution from anthropogenic activities. In this study, we quantified summer concentrations and fluxes of carbon dioxide (CO_2_), methane (CH_4_), nitrous oxide (N_2_O), and dinitrogen (N_2_), as well as several water quality parameters along the Rhine River and the Mittelland Canal, two critical inland waterways in Germany. Our main objectives were to compare GHG concentrations and fluxes along the two ecosystems and to determine the main driving factors responsible for their longitudinal GHG heterogeneities. The results indicated that the two ecosystems were sources of GHG fluxes to the atmosphere, with the Mittelland Canal being a hotspot for CH_4_ and N_2_O fluxes. We also found significant longitudinal GHG flux discontinuities along the mainstems of both ecosystems, which were mainly driven by divergent drivers. Along the Mittelland Canal, peak CO_2_ and CH_4_ fluxes coincided with point pollution sources such as a joining river tributary or the presence of harbors, while harbors and in-situ biogeochemical processes such as methanogenesis and respiration mainly explained CH_4_ and CO_2_ hotspots along the Rhine River. In contrast to CO_2_ and CH_4_ fluxes, N_2_O longitudinal trends along the two lotic ecosystems were better predicted by in-situ parameters such as chlorophyll-*a* concentrations and N_2_ fluxes. Based on a positive relationship with N_2_ fluxes, we hypothesized that in-situ denitrification was driving N_2_O hotspots in the Canal, while a negative relationship with N_2_ in the Rhine River suggested that coupled biological N_2_ fixation and nitrification accounted for N_2_O hotspots. These findings stress the need to include N_2_ flux estimates in GHG studies, as it can potentially improve our understanding of whether nitrogen is fixed through N_2_ fixation or lost through denitrification.

**Supplementary Information:**

The online version contains supplementary material available at 10.1007/s11356-024-33394-8.

## Introduction

Inland waters, comprising lotic (e.g., streams, rivers, and canals) and lentic (e.g., reservoirs, lakes, and ponds) ecosystems, are increasingly recognized as significant sources of greenhouse gases (GHGs), contributing ~ 15% (median: 8.3; interquartile range: 5.7–12.7 Pg CO_2_-eq based on a global warming potential of 100 years) to the total global carbon dioxide equivalent (CO_2_-eq) emissions (Jones et al. 2023; Lauerwald et al. 2023). In comparison, lotic ecosystems are characterized by disproportionately higher CO_2_-eq emissions (> 60%) than lentic ecosystems, linked to their close connectivity to terrestrial landscapes and their turbulent flow conditions, which favors gas evasion to the atmosphere (Raymond et al. 2013; Rocher-Ros et al. 2023; Yao et al. 2020). The GHG concentrations and fluxes of lotic ecosystems are also highly heterogeneous in space due to the variable nature of surrounding landscapes affecting local biogeochemical processes and differences in channel geomorphologies (e.g., Ho et al. 2022; Mwanake et al. 2022; Park et al. 2023; Teodoru et al. 2015), both of which may introduce significant uncertainties to GHG emission estimates from inland waters if not precisely quantified.

While research has demonstrated that headwaters, the inception points of river networks, contribute more than two-thirds of the global GHG emissions from riverine ecosystems (e.g., Li et al. 2021; Liu et al. 2022), anthropogenic activities along large rivers may modify this trend. Previous studies have shown that nutrients and carbon inputs from human-influenced landscapes to large rivers through diffuse and point sources favor in-situ GHG production processes such as nitrification, incomplete denitrification, methanogenesis, and respiration, creating GHG hotspots comparable to or higher than those from headwater streams (Borges et al. 2018; Mwanake et al. 2019; Park et al. 2018; Mwanake et al. 2023a, b). For instance, Mwanake et al. (2023a) reported higher or comparable GHG emissions from temperate rivers in Germany relative to small streams linked to inorganic nitrogen (N) and labile carbon (C) inputs from surrounding upstream cropland and urban areas. Similar findings were also found along the Seine River downstream of wastewater treatment plants (WWTPs) in large metropolitan areas in France (Marescaux et al. 2018). Apart from pollution sources related to surrounding landscapes and wastewater treatment plant effluents, the presence of harbors along large rivers, characterized by low-flow velocities, has also been shown to promote organic carbon accumulation and anoxic conditions, which favor methane (CH_4_) production hotspots through increased methanogenesis (Bussmann et al. 2022). Despite the importance of these local GHG hotspots along large rivers, field studies incorporating multiple measurements of GHG fluxes along river transects are still limited (but see Begum et al. 2021; Leng et al. 2023; Park et al. 2023), making it challenging to understand the critical drivers and magnitude of their longitudinal GHG flux heterogeneities.

Drainage ditches and canals, whose GHG dynamics are often understudied compared to rivers, have also been shown to be potent GHG hotspots of mainly CH_4_ emissions due to their low-flow velocities that provide suitable anaerobic conditions for CH_4_ production. It is estimated that these canals contribute ~ 3% of the global anthropogenic CH_4_ emissions (Peacock et al. 2021), while they can also be hotspots of nitrous oxide (N_2_O) emissions, especially when they receive N inputs from surrounding landscapes (Reay et al. 2003). Like rivers, diffuse and point pollution sources along canal ecosystems may also result in significant longitudinal GHG heterogeneities. However, these pollution effects remain uncertain, as most studies often take single reach-scale samples along canals to represent whole channel GHG magnitudes (Peacock et al. 2021).

Apart from GHG hotspots directly linked to allochthonous nutrients and carbon inputs, increased water-column primary production, especially during warm periods of the year, may also alter longitudinal GHG trends along large lotic ecosystems with relatively low water flow conditions. Although the influence of primary producers on lotic GHG concentrations remains uncertain due to the scarcity of studies with paired measurements of GHG concentrations and river metabolism rates (Battin et al. 2023), several potential mechanisms can be hypothesized linked to their effects on nutrient and carbon cycling. The decomposition of organic matter could supply fresh and more labile autochthonous organic carbon (C), favoring GHG production through C and N cycling processes. At the same time, N_2_-fixing cyanobacteria may contribute to excess mineral N, driving in-situ GHG production, particularly of N_2_O.

Conventional sampling approaches, which primarily focus on single locations along large rivers and canals, have been extensively applied to determine the magnitudes of their reach-scale GHG emissions (e.g., Mwanake et al. 2022; Peacock et al. 2021). Such approaches fail to capture the intricate nuances of large-scale longitudinal GHG dynamics along lotic ecosystems related to local GHG hotspots or coldspots (e.g., Silverthorn et al. 2023; Hotchkiss et al. 2015; Bussmann et al. 2022; Park et al. 2023; Teodoru et al. 2015), resulting in overestimation or underestimation of GHG fluxes from the entire ecosystems when the single measurements are upscaled over long distances. The lack of better spatial coverage in these large lotic ecosystems has been mainly driven by extensive resource requirements in making detailed longitudinal measurements along elongated reaches that can be hundreds of kilometers long. One approach that can overcome resource barriers is using high-precision and cost-effective sensors that simultaneously measure water quality parameters such as dissolved oxygen (DO), nutrients, and organic matter (OM) content (Bieroza et al. 2023). Combined with GHG concentration analysis, these sensors may constrict the critical drivers of longitudinal GHG flux discontinuities along lotic ecosystems, enhancing our capacity to model and manage these spatial GHG hotspots.

This study tested a novel sampling approach to quantify large-scale longitudinal GHG heterogeneities along one of the largest European rivers (Rhine River) and canal systems (Mittelland Canal) located in Germany. Within the framework of a river expedition, we explored a combined river and canal length of 632 km. Our approach encompassed longitudinal measurements of grab GHG samples (N_2_O, CO_2_, and CH_4_) and onsite sensor measurements of multiple water-physicochemical variables during the temperate summer. In addition, we also took grab samples for N_2_ concentration analysis along both lotic ecosystems, which is a proxy for major N pathways (denitrification or biological N_2_ fixation) but currently remains understudied in large lotic ecosystems.

Our main objectives were (1) to compare GHG concentrations and fluxes from the Rhine River with the Mittelland Canal system; (2) to quantify large-scale longitudinal GHG heterogeneities along the two lotic ecosystems and infer the key drivers of these longitudinal GHG trends. We hypothesized that the canal would be characterized by much higher CH_4_ and N_2_O fluxes than the river, driven by more anoxic conditions, point source inputs of nutrients, and availability of labile carbon from autotrophic processes. We also hypothesized that changes in either morphological, e.g., the existence of harbors with longer water residence times that favor internal C and N cycling or biogeochemical environmental conditions, would explain most of the longitudinal variabilities along both lotic ecosystems’ mainstems.

## Materials and methods

### Study area

**Fig. 1:**
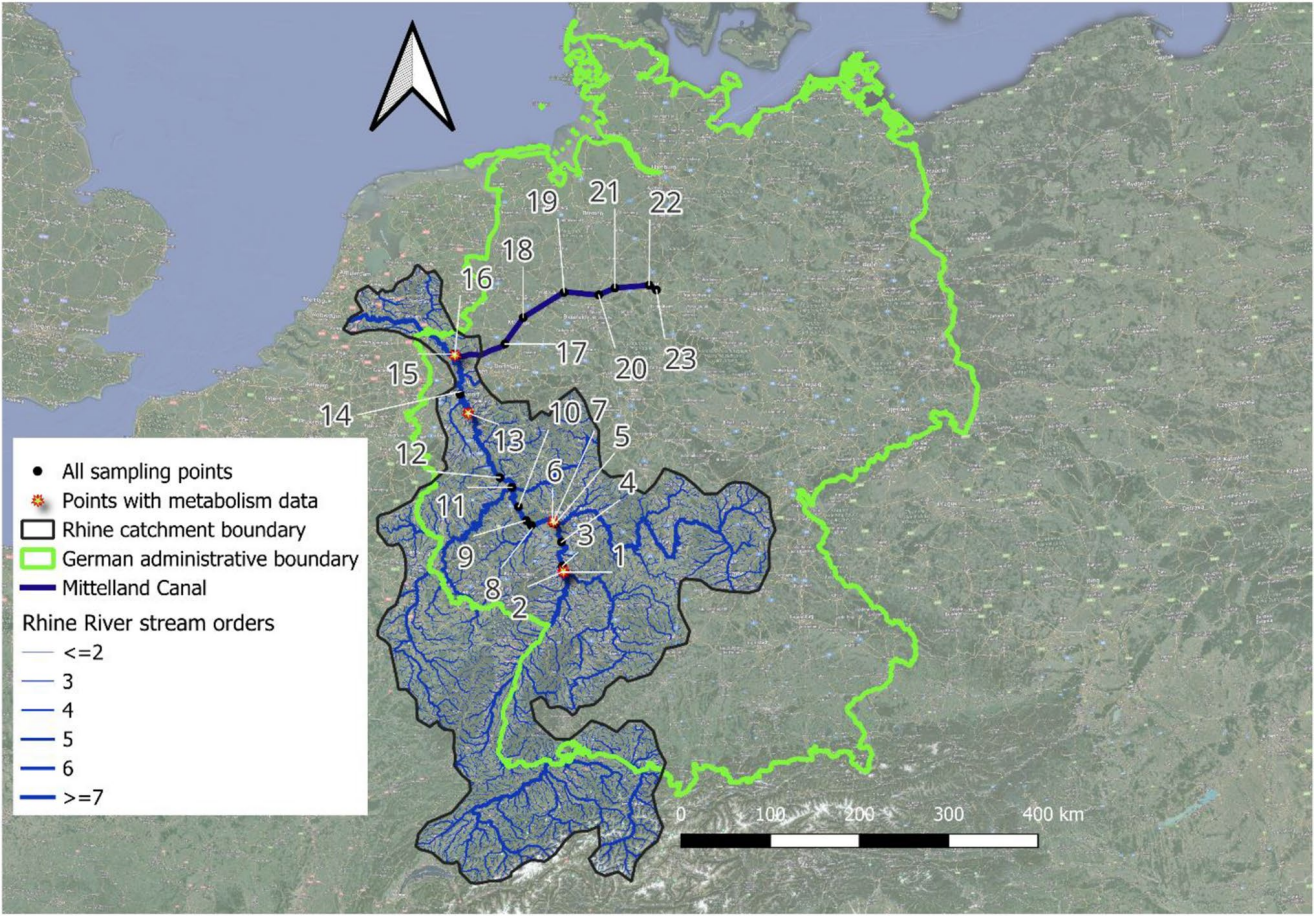
Map of the Rhine River network and the Mittelland Canal. The black dots and labels represent the 23 sampled sites with their site numbers (Table S1). The red star-shaped dots symbolize the water quality monitoring stations whose continuous oxygen and water temperature datasets were used to calculate the metabolism rates of the Rhine River. The blue lines represent the Rhine River network, with the thickness and color intensity of the lines indicating stream size/order increases. The background map is from Google Earth

The Rhine River is among the ten largest rivers in Europe, with a total length of 1233 km and a catchment size of 185,500 km^2^. The river has its sources in the Swiss Alps, enters Lake Constance on the eastern part, crosses Lake Constance via Konstanz, and leaves it on the southwestern side. From Basel onward, the Rhine River flows towards the North Sea, and from here on, the Rhine is intensively used as an inland waterway and for hydropower production, traversing multiple landscapes with several major tributaries such as the Neckar, the Main, and the Mosel joining. This study was conducted on the German side of the Rhine River catchment, one of Germany’s most important inland waterways, with a yearly transport volume of 6 million tons (Fig. 1). The first sampling point along the Rhine River was located at the midsection of the river (Mannheim, 653 km from the source), while our most downstream location was at Wesel, Germany (1023 km from the source; Table S1; Fig. 1).

The Mittelland Canal traverses Germany from west to east. It is one of the central connections of the industrialized areas in the northern Ruhr area to the large harbors of the North Sea (e.g., Rotterdam, Antwerpen, Amsterdam). In addition to the Rhine and Neckar, the Mittelland Canal is one of Germany’s most important inland waterways and has a yearly transport volume of 6 million tons. Six large locks are located between sites 16 and 23 in Friedrichsfeld, Hünxe, Dorsten, Flaesheim, Ahsen, and Datteln (Fig. 1). These locks control the water flow within the canal by pumping water in and out, assisting the movement of large ships from west to east. Apart from its use as an inland waterway, the Mittelland Canal is part of the water distribution network in Germany. Specifically, freshwater is transported from the Lippe, the lower Ruhr, and the Rhine Rivers to the east, where it is used for industries and irrigation. According to a report by a local environmental agency, the Mittelland Canal is considered highly eutrophic due to point-source inputs of nutrients from several WWTPs and rivers, such as the Weser, along its longitudinal cross section (Landesumweltamt Nordrhein-Westfalen 2002).

### Sampling strategy

In this study, sampling was performed from 12.06.2023 to 06.07.2023, mostly between 9:00 am and 5:00 pm, on the Science and Media Vessel ALDEBARAN run by the German Ocean Foundation (Table S1; Figure S1). The field campaign was part of a more extensive field expedition, which aimed at connecting actors from society, business, and politics concerning the traces of human influences on water quality and biodiversity along the Rhine. A total of 23 lotic sites were sampled, comprising 16 sites (including four harbors) along the Rhine River (386-km stretch) that represented large (> 7) stream sizes/orders and seven canal sites (including two harbors) along the Mittelland Canal (246-km stretch, Table S1; Fig. 1).

### Analysis of water and gas samples

River and canal water was collected with a bucket from a depth of ~ 1 m below the water surface and sampled for ammonium, GHGs (CO_2_, N_2_O, and CH_4_), and N_2_ concentrations. Samples for ammonium measurements were taken from the bucket and temporarily stored in a falcon tube. Analysis of ammonium (NH_4_-N) was done immediately in the field using a pHPhotoFlex Turb (WTW Germany) and the ammonium cuvette test A6/25 (173700, WTW Germany). For GHGs, triplicate samples were drawn from one bucket of water using the headspace equilibration technique (Aho and Raymond 2019). In brief, 80 mL of water was equilibrated with 20 mL of atmospheric air in a syringe after shaking for 2 min in the bucket to maintain in-situ temperatures. The headspace gas samples were transferred into pre-evacuated 10-mL glass vials for GHG concentration analysis in the laboratory using an SRI gas chromatograph (8610C, Germany) with an electron capture detector (ECD) for N_2_O and a flame ionization detector (FID) with an upstream methanizer for simultaneous measurements of CH_4_ and CO_2_ concentrations. Dissolved GHG concentrations in the river and canal water were calculated from post-equilibration gas concentrations in the headspace after correcting for atmospheric (ambient) GHG concentrations (see Mwanake et al. 2022).

Duplicate water samples for N_2_ concentration measurements were collected from the bucket in gas-tight 12-mL exetainers (Labco, UK) without air bubbles and stored in a refrigerator until analysis. In the laboratory, measurements of dissolved N_2_ were carried out on a membrane inlet mass spectrophotometer (MIMS; Bay instruments, USA) at close to in-situ temperatures (~ 20 °C) following the procedure outlined in Kana et al. (1994). In brief, the MIMS measurements involved continuous uptake of the water samples through a gas-permeable silicone membrane using a peristaltic pump and detecting N_2_ (mass 28) on a quadrupole mass spectrophotometer (Pfeiffer vacuum PrismaPlus). We used N_2_:Ar current ratios to measure N_2_ concentrations with high precision (< 0.05 CV%) (Kana et al. 1994; An et al. 2001). The MIMS setup included a liquid N_2_ trap and a reduction furnace to minimize water vapor interference and other dissolved gases on the N_2_ measurements (Kana et al. 1994).

### GHG and N_2_ flux estimation

The daily diffusive GHG and N_2_ fluxes (*F*) (moles m^−2^ d^−1^) for each of the sampling points were estimated using Fick’s Law of gas diffusion (Eq. 1), where *F* is the product of the gas transfer velocity (*k*) (m d^−1^) and the difference between the stream water concentrations (*C*_*sw*_) (moles m^−3^) and the ambient atmospheric gas concentration in water assuming equilibrium with the atmosphere (*C*_*sat*_) (moles m^−3^).

1 $$F=k ({C}_{sw}- {C}_{sat})$$

The temperature-specific gas transfer velocities (*k*) of CO_2_, CH_4_, N_2_O, and N_2_ in Eq. 1 were calculated from normalized gas transfer velocities (*k*_600_) (m d^−1^) of each site- and temperature-dependent Schmidt numbers (*Sc*) (unitless) of the respective gases (Eq. 2) (Raymond et al. 2012).

2 $$k={k}_{600} \times {\left({~}^{600}\!\left/ \!{~}_{Sc}\right.\right)}^{0.5}$$

The *k*_600_ values of each of the sampling points were predicted using Eq. 3 adapted from Raymond and Cole (2001), where *U*_10_ is the windspeed at 10 m (m s^−1^) above the water surface and 0.24 is a unit conversion factor, i.e., from cm h^−1^ gas transfer velocities to m d^−1^.

3 $${k}_{600 }=1.91{{\text{e}}}^{0.35{U}_{10}}\times 0.24$$

Site-specific windspeeds in this study were drawn from the E-OBS gridded morphological data for Europe, representing daily averages of the exact dates for each sampling point (Cornes et al. 2018; https://cds.climate.copernicus.eu/cdsapp#!/dataset/insitu-gridded-observations-europe?tab=overview, accessed on 28.02.2024). The final fluxes were then expressed in mass units by multiplying by the respective molar masses.

### Sensor measurements of water-physicochemical properties

Onsite measurements of water-physicochemical properties such as NO_3_-N, total organic carbon (TOC), total suspended solids (TSS), dissolved organic carbon (DOC), chlorophyll-*a*, UV254, and UV254f were performed simultaneously with the grab samples using a calibrated optical sensor (S::Can spectro::lyser V3, Messtechnik GmbH, Vienna, Austria). The specific ultraviolet absorbance at 254 nm (SUVA_254_, a measure of DOC quality) was calculated by dividing UV254 by DOC concentrations to estimate DOC aromaticity (e.g., Bodmer et al. 2016). Additional water parameters such as pH, conductivity, water temperature, atmospheric pressure, and dissolved oxygen were also monitored using a locally calibrated multiprobe (YSI ProDSS probe, USA). The S::Can and the YSI ProDSS probes were mounted at the back of the ship in a bucket with a volume of ~ 20 L and an overflow pipe. Water inflow was maintained by a freshwater pump from ~ 1-m depth with a water flow rate of ~ 12.7 L/min (Figure S1).

### Estimation of *k*_600_, ecosystem respiration, and gross primary production rates along the Rhine River

We used continuous hourly data (12.06.23–30.06.23) of dissolved oxygen (DO) and water temperature from four water quality monitoring stations along the Rhine River for the estimation of normalized gas transfer velocities (*k*_600_: m d^−1^), ecosystem respiration (ER), and gross primary production (GPP) rates in g O_2_ m^−2^ d^−1^. The dataset was sourced from continuous water quality monitoring stations of the NRW State Agency for Nature, Environment and Consumer Protection (LANUV) for the Wasserkontrollstation Rhine-North Kleve-Bimmen (00504) and Rhine South Bad Honnef (00103) and from the State Office for the Environment Rhineland-Palatinate (LFU RLP) for the “Rheingütestation Worms” (https://www.rheinguetestation.de) and the “Rheinwasser Untersuchungsstation Mainz-Wiesbaden” (https://www.rheinwasseruntersuchungsstation.de/). The modeling of these parameters from the hourly DO measurements was done using a Bayesian model built in the “StreamMetabolizer” R package (Appling et al. 2018). Final daily estimates of *k*_600_, GPP, and ER rates for the four sites were calculated by averaging daily estimates of the duration of our sampling campaign (22-day average). In addition, we also calculated net ecosystem production (NEP) rates as the difference between GPP and ER.

#### Statistical analysis

Analysis of variance from significant (*p*-value < 0.05) linear regression models followed by a Tukey post hoc analysis of least square means were performed to determine the combined importance of lotic ecosystem type (river and canal) and the presence of harbors on the spatial variability of water-physicochemical variables, GHG and N_2_ concentrations, and fluxes (“R stats,” “emmeans,” and “multcomp” packages in R version 4.3.2). The performances of these linear models were assessed based on the distribution of their residuals; that is, they should be normally distributed with a mean close to zero, as well as the *r*^2^ of the regressions.

Apart from broader-scale drivers of spatial variation related to ecosystem type and harbors, longitudinal trends of the water-physicochemical variables, GHG and N_2_ concentrations, and fluxes in the two lotic ecosystems were also investigated by assessing their correlative relationships with downstream distance (Pearson correlation analysis using “ggplot2” and “ggpubr” packages in R version 4.3.2). To determine the biogeochemical drivers of the longitudinal trends in GHG fluxes, several bivariate linear regression analyses were performed, and a *p*-value threshold of < 0.05 was applied to determine significant relationships (R stats package in R version 4.3.2). Bivariate relationships with *p*-values ranging from 0.05 to 0.10, which may have been insignificant due to our small sample sizes and high spatial variability in the dataset, were also considered to represent possible positive or negative trends with the GHG fluxes.

The dependent variables in the regression models were CO_2_, CH_4_, and N_2_O fluxes. The independent variables in the models were water temperature, SUVA_254_, chlorophyll-*a*, DOC, TOC, TSS, NH_4_-N, NO_3_-N, and N_2_ fluxes, which serve as direct or indirect indicators of GHG production or consumption processes. Additional independent variables such as GPP, ER, and NEP rates were used at four sites along the Rhine River where the data was available (see the “Materials and methods” section for details). Because all three GHG fluxes were not normally distributed, we transformed them using the natural logarithm to meet the normality assumption required for the regression models and also to improve model performance.

## Results

### Comparisons between the river and canal ecosystems

#### Water-physicochemical properties

**Fig. 2:**
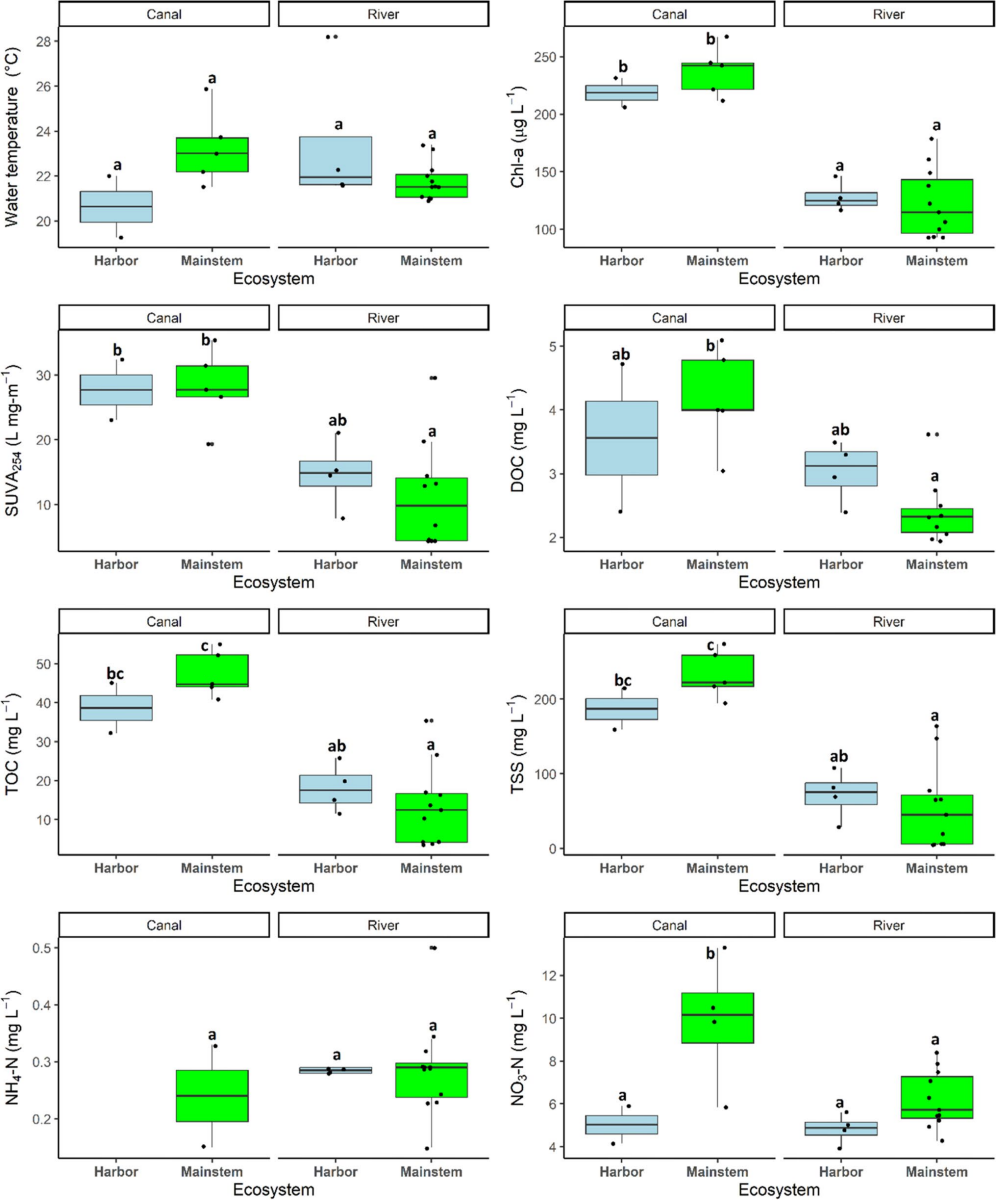
Boxplots indicating the interactive effect of lotic ecosystem type and harbor presence on the spatial variability of water-physicochemical properties. Black dots represent the individual data points for each site. The letters on top of the boxplots indicate the significant differences from Tukey post hoc analysis of least square means from the regression models (Table 1)

Summertime water-physicochemical variables along the longitudinal transects of the two lotic ecosystems varied up to an order of magnitude, with SUVA_254_ (a measure of DOC quality), chlorophyll-*a*, TOC, and TSS concentrations showing the highest variabilities, ranging from 4.34 to 35.35 L mg-m^−1^, 92.87 to 267.30 µg L^−1^, 3.56 to 55.05 mg L^−1^, and 4.64 to 273.74 mg L^−1^, respectively (Fig. 2). In contrast, water temperature and DOC concentrations had a narrower range of 19.26–28.20 °C and 1.94–5.09 mg L^−1^, respectively. Chlorophyll-*a*, DOC, SUVA_254_, TOC, and TSS were significantly higher in the Mittelland Canal than in the Rhine River (Table 1; Fig. 2).

**Table 1:** Analysis of variance results from linear regression models predicting the interactive effect of lotic ecosystem type (river vs. canal) and presence of harbors on water-physicochemical properties, GHG and N_2_ concentrations, and fluxes

		ANOVA from linear regression models		
Independent variable	df	*F*-statistic	*p*-value	*r*^2^
Water temperature (°C)	3	2.21	0.121	
Chl-*a* (μg L^−1^)	3	19.20	< 0.001	0.72
SUVA_254_ (L mg-m ^−1^)	3	7.34	0.002	0.49
DOC (mg L^−1^)	3	7.82	0.002	0.51
TOC (mg L^−1^)	3	19.50	< 0.001	0.73
TSS (mg L^−1^)	3	18.41	< 0.001	0.71
NH_4_-N (mg L^−1^)	3	0.33	0.727	
NO_3_-N (mg L^−1^)	3	7.18	0.003	0.48
CO_2_ (μmol L^−1^)	3	1.00	0.415	
CO_2_-C flux (mg m^−2^ d^−1^)	3	0.82	0.501	
CH_4_ (μmol L^−1^)	3	14.33	< 0.001	0.65
CH_4_-C flux (mg m^−2^ d^−1^)	3	12.80	< 0.001	0.62
N_2_O (μmol L^−1^)	3	1.34	0.291	
N_2_O-N (flux (mg m^−2^ d^−1^)	3	0.63	0.603	
N_2_ (μmol L^−1^)	3	0.10	0.959	
N_2_ flux (mg m^−2^ d^−1^)	3	0.19	0.903	

Only the *r*^2^ values of significant models (*p*-value < 0.05) are shown

Nitrate concentrations were generally an order of magnitude higher than NH_4_-N concentrations irrespective of ecosystem type, ranging from 3.91 to 13.27 mg L^−1^ compared to 0.15–0.50 mg L^−1^ for NH_4_-N (Fig. 2). Comparing the two lotic ecosystems, NO_3_-N concentrations were 1.4 times higher in the Mittelland Canal than in the Rhine River (Table 1; Table S2; Fig. 2). In contrast to NO_3_-N, NH_4_-N concentrations were not significantly different between the canal and the river system (Table 1; Fig. 2). The presence of harbors within both ecosystem types had no significant effects on all water quality parameters except for NO_3_-N concentrations in the Mittelland Canal, which were 2 times lower in the harbors than in the canal mainstem (Table 1; Table S2; Fig. 2).

#### Normalized gas transfer velocity, GHG and N_2_ concentrations and fluxes

The *k*_600_ values modeled from the windspeed data ranged from 0.86 to 2.21 m d^−1^ across all 23 sites. At the four stations along the Rhine River where *k*_600_ was also modeled from O_2_ data, the mean and range of these values were within the same order of magnitude as the windspeed estimates, though the O_2_-based estimates had a higher mean and were more variable than the windspeed-based estimates (O_2_ estimates: mean; 1.88, range; 1.47–2.21 m d^−1^; windspeed estimates: mean; 1.34, range; 1.23–1.38 m d^−1^).

**Fig. 3:**
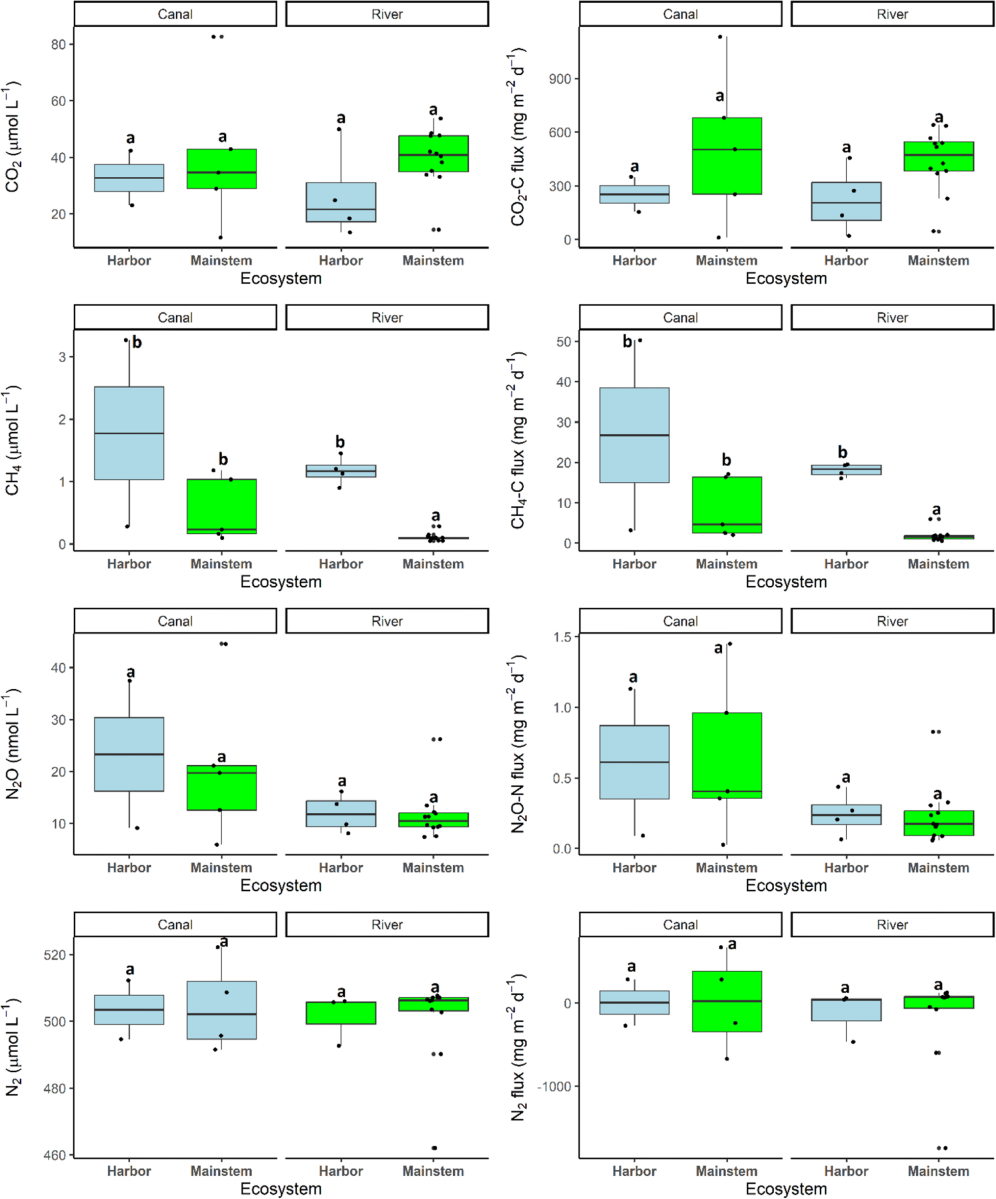
Boxplots indicating the interactive effect of lotic ecosystem type and the presence of harbors on the spatial variability of GHG and N_2_ concentrations and fluxes. Black dots represent the individual data points at each site. The letters on top of the boxplots indicate the significant differences from Tukey post hoc analysis of least square means from the regression models (Table 1)

In terms of emissions, the Rhine River and the Mittelland Canal were both sources of GHGs to the atmosphere indicated by their positive flux values (Fig. 3). Their concentrations and fluxes were also highly variable across the two ecosystems inclusive or exclusive of harbors, ranging up to two orders of magnitude, i.e., from 0.05 to 3.27 µmol L^−1^ and 0.52 to 50.29 mg m^−2^ d^−1^ for CH_4_-C, from 11.66 to 82.61 µmol L^−1^ and 10.90 to 1133.60 mg m^−2^ d^−1^ for CO_2_-C, and from 5.92 to 44.56 nmol L^−1^ and 0.03 to 1.45 mg m^−2^ d^−1^ for N_2_O-N (Fig. 3).

Methane concentrations and fluxes were ~ 5 times higher in the Mittelland Canal than in the Rhine mainstem. However, harbors along the Rhine created CH_4_ hotspots, with CH_4_ fluxes comparable to those quantified at the Mittelland Canal (Table 1; Table S2; Fig. 3). Even though ecosystem type and the presence of harbors had no significant effect on CO_2_ and N_2_O concentrations and fluxes (Table 1), the mean CO_2_ values tended to be lower in the harbors than in the mainstems of the Mittelland Canal and Rhine River, while the mean N_2_O concentrations and fluxes were ~ 2 times higher in the Mittelland Canal than in the Rhine River (Table S2; Fig. 3).

In contrast to the GHGs, N_2_ fluxes in the two lotic ecosystems indicated either sinks or sources of the gas to the atmosphere based on their positive and negative values (Table S2; Fig. 3). N_2_ concentrations and fluxes ranged from 462.08 to 522.20 µmol L^−1^ and − 1747.31 to 673.91 mg m^−2^ d^−1^, respectively (Fig. 3). While neither ecosystem type nor harbors significantly affected N_2_ concentrations and fluxes (Table 1), the highest negative N_2_ fluxes equal to biological N_2_ fixation were predominantly found in the Rhine River (Fig. 3).

#### Estimated GPP, ER, and NEP rates from continuous hourly O_2_ data

**Fig. 4:**
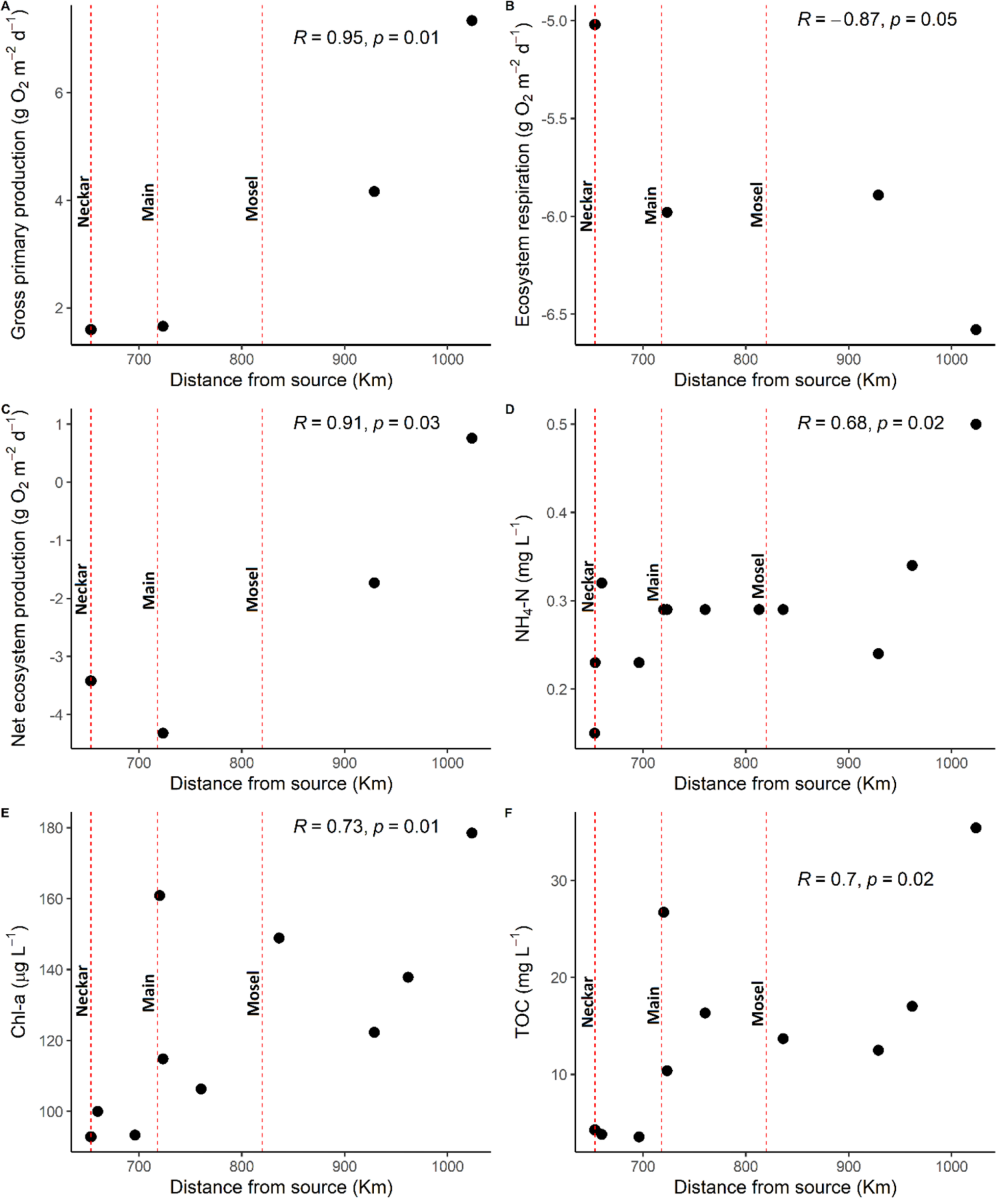
Longitudinal trends of **A**, **B**, and **C** GPP, ER, and NEP rates and **D**, **E**, and **F** NH_4_-N, chlorophyll-*a*, and TOC concentrations along the Rhine mainstem. The dotted lines on the graphs represent the inflow of major tributaries along the Rhine (Neckar, Main, Mosel). Text on the graphs shows the Pearson correlation coefficients (*r*) and *p*-value of the longitudinal trends with downstream distance

At the four sites along the mainstem of the Rhine River, the estimated daily mean GPP, ER, and NEP rates for the duration of our study ranged from 1.59 to 7.33, − 6.57 to − 5.02, and − 4.31 to 0.75 g O_2_ m^−2^ d^−1^, respectively (Fig. 4). GPP, ER, and NEP rates increased downstream, with correlation coefficients ranging from 0.87 to 0.91 (Fig. 4A, B, C). These increasing trends were also found for water-physicochemical properties such as NH_4_-N, chlorophyll-*a*, and TOC concentrations, with significant correlation coefficients ranging from 0.68 to 0.73.

#### Longitudinal GHG trends along the two lotic ecosystems not linked to harbors

**Fig. 5:**
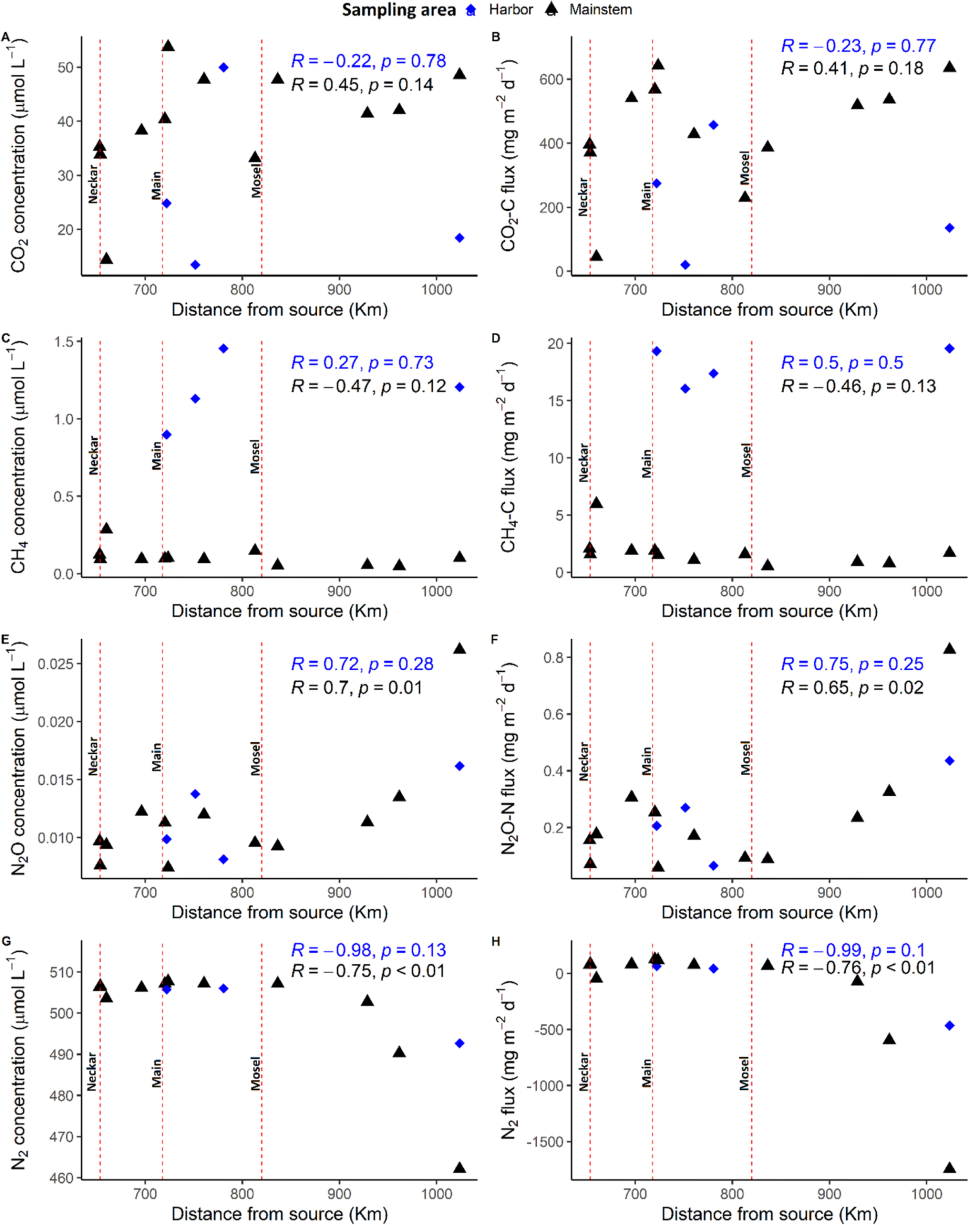
Longitudinal trends of CO_2_, CH_4_, N_2_O, and N_2_ concentrations and fluxes along the Rhine River. Blue-colored points with diamond shapes indicate the harbor sites, while black-colored points with triangle shapes indicate the sites along the mainstem. The dotted lines on the graphs represent the inflow of major tributaries along the Rhine (Neckar, Main, Mosel). Text on the graphs shows the Pearson correlation coefficients (*r*) and *p*-value of the longitudinal trends with downstream distance

The presence of harbors resulted in discontinuities in the longitudinal GHG trends along the Rhine River, particularly for CO_2_ and CH_4_ concentrations and fluxes (Fig. 5). That said, the spatial heterogeneities of the GHG fluxes along the Rhine mainstem were still significant even when harbor sites were excluded, with coefficient of variation (CV) values of 39% for CO_2_, 79% for CH_4_, and 91% for N_2_O.

Similar to the metabolism estimates and some water-physicochemical parameters, GHG concentrations and fluxes along the mainstem of the Rhine also showed correlative patterns with the distance from the Rhine’s source, albeit not always significant (Pearson correlation coefficient; *p*-value > 0.05) (Fig. 4; Fig. 5). Carbon dioxide concentrations and fluxes tended to increase downstream, with the highest value found after the confluence with the Main River, which also had elevated chlorophyll-*a* and TOC concentrations (Fig. 4E, F; Fig. 5A, B). In contrast to CO_2_, CH_4_ concentrations and fluxes decreased downstream, with the highest value found after the confluence with the Neckar River (Fig. 5C, D).

River confluences and harbors had less influence on the downstream trends of N_2_O, and thus, these trends were more unidirectional, with concentrations and fluxes showing significant increases with distance from the Rhines source (Fig. 5,E, F). The increases in N_2_O concentrations and fluxes also coincided with simultaneous increases in NH_4_-N concentrations (Fig. 4D) but were contrary to N_2_ concentration and N_2_ flux trends, which declined with the distance from the Rhines source (Fig. 5,G, H).

Compared to the Rhine River, the variation in GHG fluxes along the Mittelland Canal’s mainstem was up to 2 times higher, with CV values of 83% for CO_2_, 89% for CH_4_, and 88% for N_2_O (Figure S2). The highest CH_4_ concentrations and fluxes were found at a harbor site with low CO_2_ concentrations and fluxes, while the highest CO_2_, N_2_O, and N_2_ concentrations and fluxes were found after the confluence with the Weser River (Figure S2).

#### Biogeochemical controls on the longitudinal GHG flux trends along the Rhine River and the Mittelland Canal mainstems

We used bivariate linear regressions to reveal the most relevant biogeochemical drivers of the longitudinal GHG flux trends along the Rhine River and the Mittelland Canal mainstems. The regressions were based on GHG flux relationships with in-situ water**-**physicochemical variables and N_2_ fluxes, as well as GPP, ER, and NEP rates available for the Rhine River (Table 2). At the Rhine, CO_2_ fluxes were significantly positively related to ER rates, while CH_4_ fluxes showed decreasing trends with the increase in NO_3_-N concentrations (Table 2). In contrast to CO_2_ and CH_4_, the increasing trend of N_2_O fluxes with distance from the source was significantly predicted by most of the water-physicochemical properties and process rates that showed similar unidirectional trends (Table 2; Fig. 4). N_2_O fluxes were positively related to TOC and TSS concentrations and GPP and NEP rates and negatively related to N_2_ fluxes (Table 2). N_2_O fluxes also showed an increasing trend with NH_4_-N concentrations, even though the relationship was insignificant (*p*-value 0.06).

**Table 2:** Summary results of bivariate linear regression models indicating the relationship of water-physicochemical properties, N_2_ fluxes, GPP, ER, and NEP rates with GHG fluxes (transformed with the natural logarithm) in the Rhine River and Mittelland Canal mainstem (harbors not included)

Dependent and independent variables	Rhine			Canal		
	*p*-value	Slope	*r*^2^	*p*-value	Slope	*r*^2^
**Ln CO**_**2**_** flux (mg m**^−2^** d**^−1^**)**						
ER (g O_2_ m^−2^ d^−1^)	0.02	− 0.36	0.88			
**Ln CH**_**4**_** flux (mg m**^−2^ **d**^−1^**)**						
NO_3_-N (mg L^−1^)	0.08	− 0.26	0.30			
N_2_ flux (mg m^−2^ d^−1^)				0.10	2.38	0.80
**Ln N**_**2**_**O flux (mg m**^−2^** d**^−1^**)**						
DOC (mg L^−1^)	0.02	− 0.89	0.52			
TOC (mg L^−1^)	0.04	0.05	0.38			
TSS (mg L^−1^)	0.05	0.01	0.36			
NH_4_-N (mg L^−1^)	0.06	4.96	0.30			
GPP (g O_2_ m^−2^ d^−1^)	0.02	0.40	0.88			
NEP (g O_2_ m^−2^ d^−1^)	0.01	0.51	0.93			
N_2_ flux (mg m^−2^ d^−1^)	0.02	− 0.37	0.47	0.05	1.83	0.90

Only significant relationships (*p*-value < 0.05) and those with either positive or negative trends (*p*-values 0.05–0.10) with the GHG fluxes are shown. The complete table with all predictor variables and the *p*-values of the relationships is included in the supplementary materials (Table S3)

Contrary to the Rhine River, the longitudinal GHG variability in the Mittelland Canal mainstem was much less correlated with water-physicochemical parameters. CO_2_ fluxes showed no significant relationship with water-physicochemical properties and N_2_ fluxes, while both CH_4_ and N_2_O fluxes only showed significant positive relationships or just trends with N_2_ fluxes (Table 2).

## Discussion

This study provided a spatially explicit dataset covering GHG concentration measurements and estimated fluxes of GHGs and N_2_, accompanied by measurements of various water-physicochemical properties in the Rhine River and Mittelland Canal, thus enabling the determination of important drivers of their longitudinal GHG flux heterogeneities. We found that summertime GHG fluxes in these two lotic ecosystems were always positive, contributing to the growing evidence that lotic ecosystems are significant sources of GHGs to the atmosphere (Lauerwald et al. 2023; Peacock et al. 2021; Rocher-Ros et al. 2023).

In agreement with our hypothesis, the Mittelland Canal, which has been reported to receive several inflows from WWTPs and river tributaries (Landesumweltamt Nordrhein-Westfalen 2002), had higher N_2_O and CH_4_ fluxes than the Rhine River. We contend that these inflows likely contributed to elevated nutrients and labile organic carbon concentrations, which fueled in-situ N_2_O and CH_4_ production processes or directly added externally sourced dissolved N_2_O and CH_4_, similar to what was found in other studies (Brown et al. 2023; Mwanake et al. 2023a; Zhang and Chadwick 2022).

The comparison of both lotic ecosystems showed that divergent drivers controlled longitudinal heterogeneities in the GHG fluxes. Spatial variability of GHG fluxes along the Rhine River was linked to harbors and site-specific biogeochemical process rates, agreeing with findings from previous studies (Hotchkiss et al. 2015; Bussmann et al. 2022; Park et al. 2023; Mwanake et al. 2023a). For example, harbors at the Rhine River led to local hotspots of CH_4_ emission, which we linked to longer water residence times promoted by the still waters that favor organic matter and sediment accumulation, resulting in anoxic conditions and methane production. At the same time, the downstream increasing trends of N_2_O and CO_2_ concentrations and fluxes were linked to either autotrophic or heterotrophic source processes inferred from in-situ estimates of N_2_ fluxes, as well as GPP, ER, and NEP rates.

In contrast to the Rhine River, most of the longitudinal GHG trends in the canal were challenging to predict from changes in in-situ water-physicochemical properties. This finding suggested that other factors not quantified in this study, such as point-pollution sources of GHGs, nutrients, and organic carbon, may have significantly controlled longitudinal GHG heterogeneities along the Mittelland Canal. Nevertheless, we did find that 90% of the N_2_O flux spatial variability in the canal ecosystem was linked to N_2_ emissions. This result implied that denitrification was an essential source of N_2_O in the canal, opposite to what we found in the Rhine River, where coupled N_2_ fixation and nitrification inferred from concurrent negative N_2_ fluxes and elevated NH_4_-N concentrations may have accounted for N_2_O flux hotspots. Overall, our findings suggested that single GHG measurements along large lotic ecosystems, which ignore local GHG hotspots linked to the presence of harbors, point pollution sources, or biogeochemical cycling, may result in significant uncertainties in entire ecosystem GHG emission estimates. This conclusion was particularly true for the canal ecosystem, which was characterized by higher and more spatially variable GHG concentrations and fluxes than the river ecosystem.

### Longitudinal discontinuities in CO_2_ fluxes linked to harbors, point pollution sources, and metabolism rates

Carbon dioxide concentrations and fluxes quantified in this study are within the range of those quantified from other studies in temperate ecosystems, as well as the global estimates (Lauerwald et al. 2015; Raymond et al. 2013), but are much lower than those quantified from heavily polluted Asian rivers (Begum et al. 2021). Previous studies in riverine ecosystems have found CO_2_ uptake during the temperate summer due to autotrophic CO_2_ fixation (Gómez-Gener et al. 2021). However, this study’s daytime CO_2_ fluxes along the Rhine River were surprisingly positive throughout the summer campaign, suggesting that CO_2_ production via ER outweighed CO_2_ consumption via gross primary production (GPP). This conclusion is supported by the net ecosystem production (NEP) estimates from four sites along the Rhine that were primarily negative, indicating a net CO_2_ loss to the atmosphere.

Like the Rhine River, the Mittelland Canal also had positive CO_2_ fluxes, albeit slightly higher than the Rhine River. Judging by the higher carbon availability in the canal, one may assume that net in-situ heterotrophic processes were driving CO_2_ fluxes, similar to what we inferred for the Rhine. NEP estimates were unavailable for the Mittelland Canal, which did not allow us to link net heterotrophy to CO_2_ fluxes. However, based on a previous environmental report that indicated several inflows of treated wastewater or river tributaries into the Mittelland Canal (Landesumweltamt Nordrhein-Westfalen 2002), we hypothesized that the CO_2_ fluxes in the canal are likely driven by external dissolved CO_2_ supplies from these point-sources rather than internal production (e.g., Mwanake et al. 2023a). While it was difficult to indicate the contributions of WWTPs, we found that peak CO_2_ fluxes in the Mittelland Canal coincided with the inflow of the Weser River, agreeing with our hypothesis that external inflows of CO_2_ from a point pollution source likely contributed to the elevated CO_2_ fluxes in the canal.

Along the two large lotic ecosystems, longitudinal discontinuities in CO_2_ concentrations and fluxes were collectively linked to harbors, point pollution sources, and metabolism rates, similar to several other lotic studies (Begum et al. 2021; Deemer et al. 2016; Mwanake et al. 2023a). Within the Rhine River, harbor areas resulted in lower CO_2_ concentrations and fluxes than the river mainstem, which we attributed to their morphology that favors low-flow conditions, accumulation of organic matter, and anoxic conditions that limit aerobic respiration rates. This conclusion is further supported by the slightly higher DOC, TOC, and TSS concentrations in the harbors than in the mainstem of the Rhine.

Besides morphological controls, biogeochemical rates supported CO_2_ hotspots along the Rhine’s mainstem. Previous fluvial studies have linked increased ER rates with organic carbon concentrations (Mulholland et al. 2001; Piatka et al. 2021; Stelzer et al. 2003). This study found a concurrent increase in downstream TOC and CO_2_ fluxes, suggesting that the increasing TOC favored instream CO_2_ production via favored ER rates. Furthermore, our study revealed a positive correlation between ER and CO_2_ fluxes, which accounted for up to 88% of the downstream increasing trend that we found for the fluxes, strengthening this argument. In contrast to the Rhine River, the longitudinal trends in the Mittelland Canal were not predicted by any of our measured water-physicochemical properties, with peak CO_2_ fluxes coinciding with the inflow of the Weser River.

### Longitudinal discontinuities in CH_4_ fluxes linked to harbors and methanogenesis

Like CO_2_, riverine CH_4_ concentrations and fluxes quantified here were mainly within the range of global estimates (Rocher-Ros et al. 2023). However, those quantified in the canal ecosystem were primarily at the higher end of the estimates. These findings strengthen the idea that canal ecosystems are hotspots for CH_4_ emissions, similar to what has been found in previous studies (Peacock et al. 2021; Rocher-Ros et al. 2023).

The longitudinal variability of CH_4_ concentrations and fluxes in the Rhine River was linked to both harbors and biogeochemical drivers. Contrary to CO_2_, harbor areas were hotspots for CH_4_ fluxes, a finding agreeing with our earlier hypothesis that low-flow conditions and high organic matter accumulation in these areas likely favor anoxic conditions suitable for methanogenesis. A similar longitudinal study along the Elbe River also found higher CH_4_ fluxes in harbor areas, alluding to similar controls of longer water residence time and high sediment and organic matter conditions favorable for methane production (Bussmann et al. 2022).

Methane variability was attributed to variable production rates via methanogenesis along the Rhine’s mainstem. Several studies have shown that negative correlations between NO_3_-N and CH_4_ imply methane production via methanogenesis, as NO_3_-N inhibits the processes as a terminal electron acceptor (TEA) (Baulch et al. 2011; Schade et al. 2016). Our study found a decreasing trend of CH_4_ fluxes with NO_3_-N concentrations (*p*-value = 0.08), pointing out that methane production hotspots via methanogenesis along the Rhine may occur in areas with low TEA, such as NO_3_-N. Unlike CO_2_, CH_4_ fluxes along the canal mainstem showed an increasing trend with N_2_ fluxes (*p*-value = 0.10), suggesting that anoxic conditions that favor denitrification may also account for CH_4_ production hotspots via methanogenesis.

### Longitudinal discontinuities in N_2_O fluxes linked to N transformation processes

Like CO_2_ and CH_4_, riverine N_2_O fluxes fell within the global range of previous estimates from large rivers (Hu et al. 2016), with those from the Mittelland Canal comparable to N_2_O flux values from drainage ditches draining cropland landscapes ladened by high NO_3_ concentrations (Reay et al. 2003). The high NO_3_ conditions in the Mittelland Canal relative to the Rhine River resulted in more than double the N_2_O fluxes in the latter ecosystem.

Several studies in lotic ecosystems have linked high NO_3_ concentrations to elevated N_2_O production from incomplete denitrification (Andrews et al. 2021; Beaulieu et al. 2008; Mwanake et al. 2019). However, very few studies have connected N_2_O fluxes with direct measures of denitrification (e.g., Beaulieu et al. 2011), with most of the above studies inferring N_2_O sourced from denitrification based on positive correlations with NO_3_ concentrations. Such inferences may be misleading, as N_2_O production from nitrification may also have a similar positive relationship with NO_3_, the end product of nitrification. When looking at the drivers of the significant longitudinal heterogeneities in N_2_O fluxes along the Mittelland Canal, we did find direct evidence of the critical role of denitrification in its production. This evidence was based on the significant positive relationship between N_2_O and N_2_ fluxes, as positive N_2_ fluxes are solely linked to the denitrification process and hence can be used to quantify its rates (Chen et al. 2014; McCutchan et al. 2003; Ritz et al. 2018; Wang et al. 2018).

In contrast to the canal ecosystem, longitudinal N_2_O hotspots downstream of the Rhine River were linked to processes other than denitrification. High N_2_ fixation rates by cyanobacteria, autotrophic and heterotrophic diazotrophs, which supply fresh and bioavailable N, have been previously reported in freshwater environments (Geisler et al. 2022; Riemann et al. 2022). Within rivers, several studies have linked measures of N_2_ undersaturation to significant rates of N_2_ fixation by diazotrophs (Wang et al. 2021; Wu et al. 2013). This study found substantial downstream increases in N_2_ uptake of up to 1747.31 mg m^−2^ d^−1^, which more than doubled instream N_2_O fluxes. Like N_2_O, NH_4_-N, chlorophyll-*a*, and TOC concentrations, GPP and NEP rates also increased downstream of the Rhine. These findings implied significant N_2_ fixation, possibly providing the NH_4_-N required for N_2_O production via nitrification. Such significant N_2_ fixation rates have been previously reported in similar highly urbanized rivers in Beijing, with an N_2_ undersaturation of up to 88% (Wang et al. 2021). We also found significant positive relationships of N_2_O with TOC concentrations, GPP, and NEP rates and their antagonistic relationship with N_2_ fluxes, further supporting this argument. Cumulatively, these findings stress the need to include N_2_ flux estimates in quantifying GHG from lotic ecosystems, as it has the potential to determine whether nitrogen is fixed through N_2_ fixation or lost through denitrification, with severe consequences to both N and C cycling that results in the production of GHG from these ecosystems.

## Conclusion

Our study indicated that local hotspots of biogeochemical C and N cycling, point pollution sources, and harbors controlled the longitudinal GHG heterogeneities in two lotic ecosystems, albeit with different levels of importance depending on ecosystem type. These findings were primarily informed by combining GHG and N_2_ concentration measurements and flux estimates with onsite water quality estimates from sensors, suggesting that such a methodology is most suitable for quantifying the drivers of large-scale GHG flux heterogeneities. However, our study was conducted only during the summer season, and as such, seasonal differences in discharge, temperature, and GHGs were not considered, which may additionally alter longitudinal GHG trends along the two lotic ecosystems as has been found in previous studies (Aho et al. 2022; Herreid et al. 2021; Mwanake et al. 2022, 2023a). For example, on the one hand, heavy precipitation-driven discharge events may tend to reduce local GHG hotspots due to shorter water residence times that inhibit internal GHG production processes but may increase the overall fluxes due to elevated gas transfer velocities and external GHG inputs, particularly in agricultural-dominated catchments (e.g., Mwanake et al. 2022, 2023b). On the other hand, cold seasons may promote local GHG hotspots as microbial communities may face less competition for nutrients and organic carbon from plants (e.g., Herreid et al. 2021; Mwanake et al. 2023a) .

## Supplementary Information

Below is the link to the electronic supplementary material.

Supplementary file1 (DOCX 1432 KB)

## Data Availability

All data and data analyses codes of this manuscript will be made available upon request to the corresponding author.
