# Supplementary material for: Divergent drivers of the spatial variation in greenhouse gas concentrations and fluxes along the Rhine River and the Mittelland Canal in Germany
Source: Environ Sci Pollut Res Int. 2024 Apr 22;31(22):32183–99. doi: 10.1007/s11356-024-33394-8 (PMC11512915; doi:10.1007/s11356-024-33394-8)
Supplement: Supplementary file 1 — Supplementary file1 (DOCX 1432 KB) [file 11356_2024_33394_MOESM1_ESM.docx]

# Supplementary information

Title: Divergent drivers of the spatial variation in greenhouse gas concentrations and fluxes along the Rhine River and the Mittelland Canal in Germany

**Authors:** Ricky Mwangada Mwanake*^a^ (email: ricky.mwanake2@kit.edu), Hannes Klaus Imhof ^a^ (email; hannes.imhof@kit.edu), Ralf Kiese ^a^ (email; ralf.kiese@kit.edu)

^a^Karlsruhe Institute of Technology, Institute for Meteorology and Climate Research, Atmospheric Environmental Research (IMK-IFU), Kreuzeckbahnstrasse 19, Garmisch-Partenkirchen 82467, Germany

*Corresponding author: Ricky Mwangada Mwanake

Table S1:  Descriptions of the sampling sites, including name, date of sampling, channel morphology, and coordinates of sampling sites.

|  |  |  |  |  |  | **Channel Morphology** |
| --- | --- | --- | --- | --- | --- | --- |
| **Site ID** | **Site Name** | **Longitude** | **Latitude** | **Ecosystem** | **Sampling date** |  |
| 1 | Rhine, before Neckar | 49.5079 | 8.4373 | River | 12.06.2023 | Mainstem |
| 2 | Rhine, after Neckar | 49.5138 | 8.4350 | River | 12.06.2023 | Mainstem |
| 3 | Rhine | 49.5685 | 8.4173 | River | 13.06.2023 | Mainstem |
| 4 | Rhine | 49.8095 | 8.3949 | River | 13.06.2023 | Mainstem |
| 5 | Rhine before Main | 49.9945 | 8.2912 | River | 13.06.2023 | Mainstem |
| 6 | Mainz harbor | 50.0002 | 8.2502 | River | 14.06.2023 | Harbor |
| 7 | Rhine | 50.0148 | 8.2596 | River | 14.06.2023 | Mainstem |
| 8 | Bingen harbor | 49.9703 | 7.9194 | River | 14.06.2023 | Harbor |
| 9 | Rhine | 50.0177 | 7.8393 | River | 15.06.2023 | Mainstem |
| 10 | St Goar harbor | 50.1540 | 7.7088 | River | 15.06.2023 | Harbor |
| 11 | Rhine | 50.3470 | 7.5977 | River | 16.06.2023 | Mainstem |
| 12 | Rhine | 50.4423 | 7.4000 | River | 17.06.2023 | Mainstem |
| 13 | Rhine | 51.0742 | 6.8592 | River | 20.06.2023 | Mainstem |
| 14 | Rhine | 51.2592 | 6.7168 | River | 21.06.2023 | Mainstem |
| 15 | Wesel harbor | 51.6618 | 6.5873 | River | 21.06.2023 | Harbor |
| 16 | Rhine | 51.6503 | 6.6025 | River | 23.06.2023 | Mainstem |
| 17 | Mitelland Canal | 51.7850 | 7.4066 | Canal | 23.06.2023 | Mainstem |
| 18 | Mitelland Canal | 52.0562 | 7.6894 | Canal | 26.06.2023 | Mainstem |
| 19 | Bad  Essen harbor | 52.3223 | 8.3495 | Canal | 27.06.2023 | Harbor |
| 20 | Mitelland Canal | 52.3018 | 8.9190 | Canal | 27.06.2023 | Mainstem |
| 21 | Mitelland Canal | 52.3743 | 9.1819 | Canal | 28.06.2023 | Mainstem |
| 22 | Hannover harbor | 52.4056 | 9.7464 | Canal | 29.06.2023 | Harbor |
| 23 | Mitelland Canal | 52.3571 | 9.8675 | Canal | 29.06.2023 | Mainstem |

Table S2:  Summary statistics outlining the mean ± SE values for all quantified parameters in this study

|  | **Mean ± SE** | | | |
| --- | --- | --- | --- | --- |
| **Parameter** | **Harbor Canal** | **Harbor River** | **Mainstem Canal** | **Mainstem River** |
| Water temperature (°C) | 20.64 ± 1.37 | 23.42 ± 1.6 | 23.26 ± 0.75 | 21.76 ± 0.24 |
| SUVA_254_ (L mg-m ^-1^) | 27.69 ± 4.67 | 14.67 ± 2.7 | 28.08 ± 2.67 | 11.42 ± 2.41 |
| Chl-a (μg L^-1^) | 218.67 ± 12.72 | 128.12 ± 6.4 | 237.52 ± 9.69 | 122.62 ± 8.65 |
| DOC (mg L^-1^) | 3.56 ± 1.16 | 3.03 ± 0.24 | 4.18 ± 0.36 | 2.4 ± 0.14 |
| TOC (mg L^-1^) | 38.67 ± 6.42 | 18.15 ± 3.1 | 47.38 ± 2.69 | 13.45 ± 2.96 |
| TSS (mg L^-1^) | 186.88 ± 27.86 | 71.77 ± 16.51 | 233.34 ± 14.47 | 55 ± 16.43 |
| NH_4_-N (mg L^-1^) |  | 0.29 ± 0 | 0.24 ± 0.06 | 0.29 ± 0.02 |
| NO_3_-N (mg L^-1^) | 5.03 ± 0.87 | 4.82 ± 0.35 | 9.86 ± 1.37 | 6.2 ± 0.38 |
| GPP (g O_2_ m^-2^ d^-1^) |  |  |  | 3.27 ± 0.73 |
| ER (g O_2_ m^-2^ d^-1^) |  |  |  | -5.7 ± 0.19 |
| NEP (g O_2_ m^-2^ d^-1^) |  |  |  | -2.43 ± 0.58 |
| CO_2_ (μmol L^-1^) | 32.71 ± 9.62 | 26.67 ± 8.1 | 40.13 ± 11.79 | 39.7 ± 2.95 |
| CH_4_ (μmol L^-1^) | 1.77 ± 1.49 | 1.17 ± 0.11 | 0.54 ± 0.23 | 0.11 ± 0.02 |
| N_2_O (μmol L^-1^) | 0.02 ± 0.01 | 0.01 ± 0 | 0.02 ± 0.01 | 0.01 ± 0 |
| N_2_ (μmol L^-1^) | 503.45 ± 8.83 | 501.47 ± 3.81 | 504.53 ± 6.19 | 500.63 ± 3.96 |
| CO_2_ flux (mg m^-2^ d^-1^) | 251.86 ± 96.91 | 221.8 ± 93.97 | 516.66 ± 191.35 | 440.81 ± 50.03 |
| CH_4_ flux (mg m^-2^ d^-1^) | 26.72 ± 23.57 | 18.07 ± 0.83 | 8.53 ± 3.38 | 1.79 ± 0.41 |
| N_2_O flux (mg m^-2^ d^-1^) | 0.61 ± 0.52 | 0.24 ± 0.08 | 0.64 ± 0.25 | 0.23 ± 0.06 |
| N_2_ flux (mg m^-2^ d^-1^) | 8.68 ± 279.16 | -119.28 ± 199.8 | 12.6 ± 263.76 | -166.60 ± 162.68 |

Table S3: Complete summary results of bivariate linear regression models indicating the relationship of water physicochemical properties, N_2_ fluxes, GPP, ER, and NEP rates with GHG fluxes in the Rhine River and Mittelland Canal mainstem (Harbors not included).

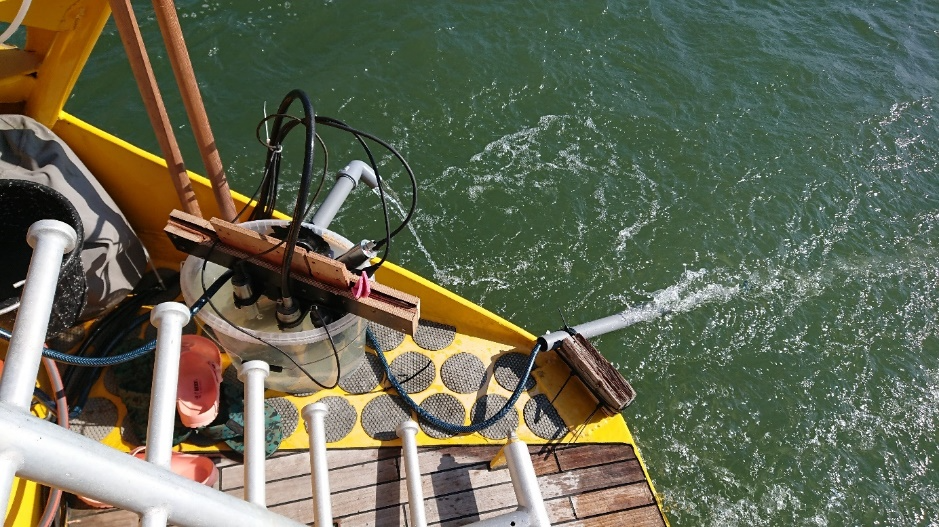


**Figure S1.** Mounting position of the bucket at the stem of the ALDEBARAN.


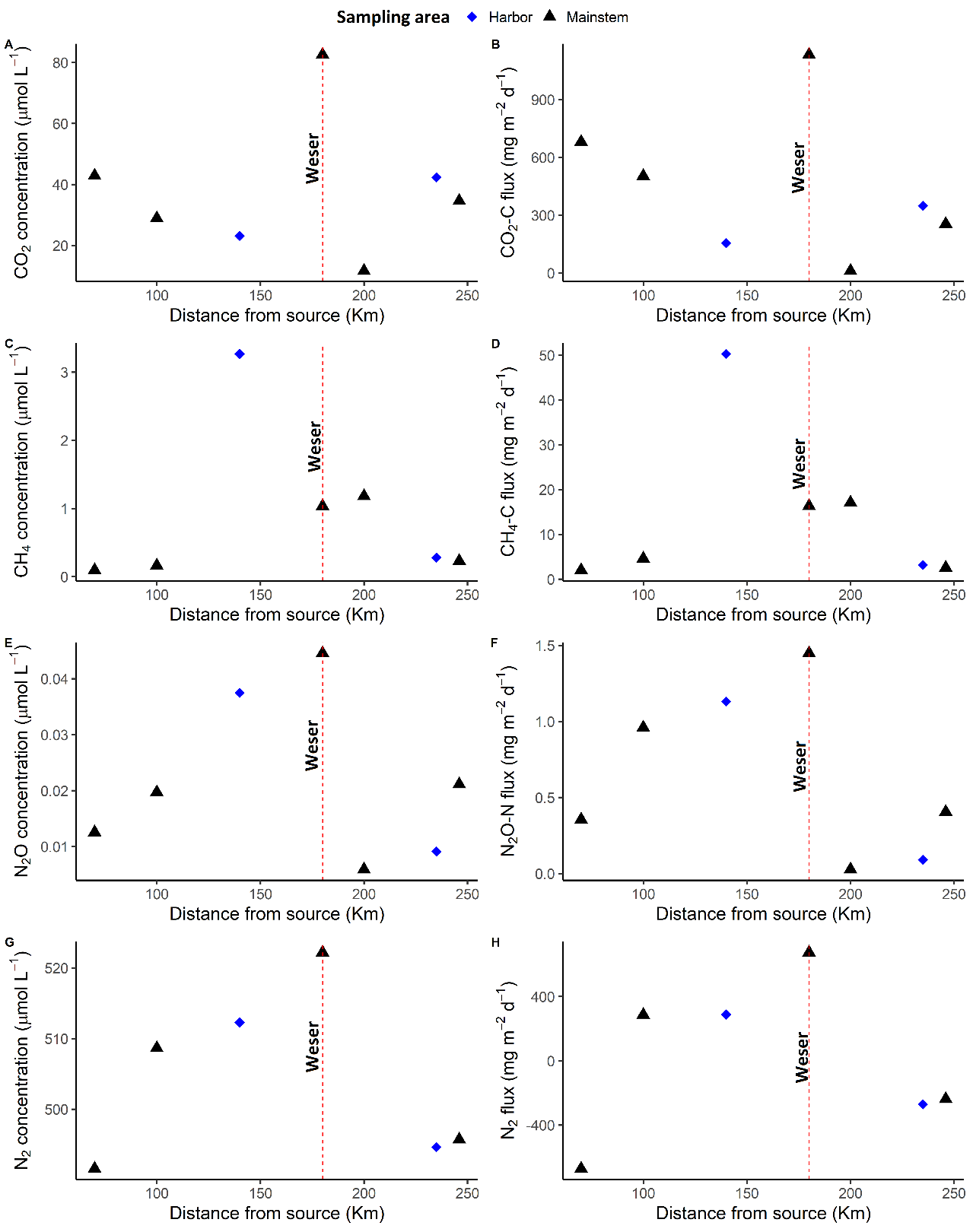


**Figure S2:** Longitudinal trends of GHG and N_2_ concentrations and fluxes along the Mittelland canal. Blue-colored points with diamond shapes indicate harbor sites, while black-colored points with triangle shapes indicate sites along the mainstem. The dotted red line indicates the inflow of the Weser River.
